# Angular-Substituted [1,4]Thiazino[3,4-a]Isoquinolines: Biological Evaluation and In Silico Studies on DPP-IV Inhibition

**DOI:** 10.3390/ijms252111753

**Published:** 2024-11-01

**Authors:** Aleksandar Pashev, Valentin Petrov, Aleksandrina Pesheva, Lidiya Petrova, Kalina Ilieva, Galya Stavreva, Milena Atanasova, Diana Cheshmedzhieva, George Altankov, Teodora Aleksandrova

**Affiliations:** 1 Faculty of Pharmacy, Medical University Pleven, 1. St. Kliment Ohridski Str., 5800 Pleven, Bulgaria; vali.petrov@abv.bg (V.P.); al.ppesheva@abv.bg (A.P.); drstavreva@yahoo.com (G.S.); teodora.aleksandrova@mu-pleven.bg (T.A.); 2 Faculty of Medicine, Medical University Pleven, 1. St. Kliment Ohridski Str., 5800 Pleven, Bulgaria; lidiia.petrova-trifonova@mu-pleven.bg (L.P.); kalina.st.ilieva@gmail.com (K.I.); milenaar2001@yahoo.com (M.A.); 3 Faculty of Chemistry and Pharmacy, Sofia University “St. Kliment Ohridski”, 1. James Bourchier Blvd., 1164 Sofia, Bulgaria; dvalentinova@chem.uni-sofia.bg; 4 Research Institute, Medical University Pleven, 5800 Pleven, Bulgaria; altankov@abv.bg

**Keywords:** [1,4]thiazino[3,4-a]isoquinolines, DPP-IV inhibitors, type 2 diabetes, cytotoxicity, molecular docking, DFT

## Abstract

Recent studies have discovered that aryl-substituted pyrido[2,1-a]isoquinolines have the potential to be highly active DPP IV inhibitors. In previous studies, we reported a novel synthetic approach for the construction of their sulfur-containing bioisosteric [1,4]thiazino[3,4-a]isoquinolines analogues, incorporating an additional aryl substituent. The present study aims to investigate the DPP IV inhibitory activity and cytotoxicity of the synthesized molecules by in vitro assay. The geometry optimization and molecular docking of the synthesized compounds were used to determine their binding modes to the active site of DPP IV. The docking analysis revealed that the energy-minimized poses of the studied compounds are close to the most important selectivity cliffs for DPP IV inhibition, forming hydrogen bonds and hydrophobic interactions with them. These results can be considered as a preliminary step towards further structural activity modifications.

## 1. Introduction

Dipeptidyl peptidase IV (DPP-IV) is a highly specific serine protease enzyme that has been widely explored for its potential to treat chronic metabolic type 2 diabetes mellitus (T2DM) [1,2,3,4,5,6]. Important endogenous physiological substrates, which are known to be cleaved by DPP-IV in vivo, are glucose-dependent insulinotropic polypeptide (GIP) and glucagon-like peptide-1 (GLP-1). These polypeptide hormones are released in the small intestine in response to the ingestion of carbohydrates, thus stimulating insulin secretion from the pancreas [6,7]. Subjects with T2DM exhibit a resistance towards the actions of GIP; therefore, this hormone is not a reliable glucose-lowering agent in such a pathological condition [8]. On the other hand, inhibition of DPP-IV in T2DM patients results in increased incretin levels of GLP-1, which, in turn, inhibits glucagon release, enhances insulin secretion, and improves glucose tolerance [9]. Recent findings suggest that DPP-IV inhibitors can also be used for the treatment of coronavirus disease 2019 (COVID-19) [10,11,12]. These findings have given rise to a drug class of DPP IV-inhibiting compounds that emerged on the market for treating type 2 diabetes [13,14,15,16,17,18,19,20].

**Figure 1:**
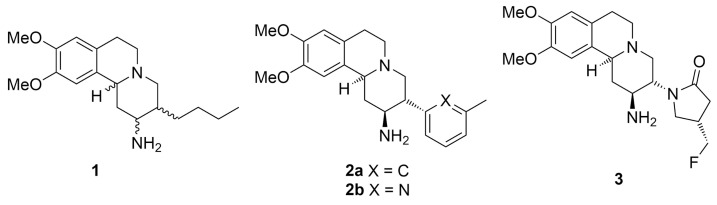
DPP-IV inhibitors incorporating pyrido[2,1-a]isoquinoline ring system.

Twelve DPP-IV inhibitors (sitagliptin, vildagliptin, saxagliptin, alogliptin, linagliptin, teneligliptin, gemigliptin, anagliptin, trelagliptin, evogliptin, omarigliptin, and denagliptin) were approved by the FDA and other regulatory agencies for clinical use, and many more are still in various phases of clinical studies [21,22,23]. Each inhibitor possesses its own unique core structure and pharmacophores, which suggests that structurally diverse molecules can interact strongly with the enzyme. Recent studies have discovered that aryl-substituted pyrido[2,1-a]isoquinolines of type **1**–**3** (Figure 1) have the potential to be highly active DPP-IV inhibitors [24,25,26]. Further evolution of this class has led to the discovery of carmegliptin **3**—a highly potent DPP-IV inhibitor that has reached phase 2 in clinical studies [25].

However, the molecular design and structural optimization of the previously reported pyrido[2,1-a]isoquinolines were directed at achieving a better occupancy of the hydrophobic S1 pocket in the enzyme active site using different acyclic and cyclic substituents [14]. This leaves plenty of unexplored possibilities for enhancing the inhibitory potential of prospective DPP-IV inhibitors featuring this core structure.

**Figure 2:**
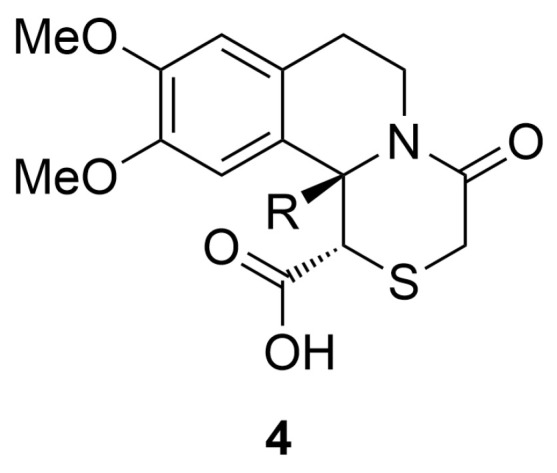
[1,4]thiazino[3,4-a]isoquinolines, used in this study.

This study aims to investigate the DPP-IV inhibitory activity and cytotoxicity of previously synthesized substituted [1,4]thiazino[3,4-a]isoquinolines derivatives featuring an angular alkyl or aryl substituent **4a**–**g** (Figure 2) [27,28]. The interaction modes of the synthesized compounds into the active site of DPP IV were further analyzed using in silico computational methods.

## 2. Results

**Scheme 1:**
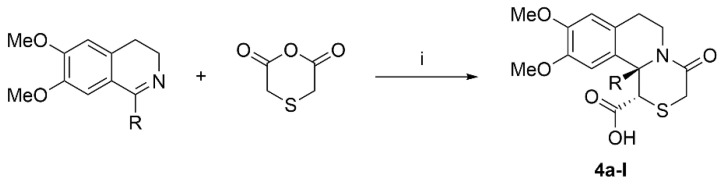
Synthesis of target compounds. Reagents and conditions: (i) xylene, inert atmosphere, 120 °C, 2–6 h.

A series of twelve aryl-substituted [1,4]thiazino[3,4-a]isoquinolines **4** analogues were previously synthesized in our lab using a novel synthetic approach between 3,4-dihidroisoquinolines and thiodiacetic anhydride (Scheme 1) [27,28]. The compounds were isolated and purified by column chromatography and subsequent recrystallization.

### 2.1. DPP-IV Inhibitory Activity

The inhibitory activity versus human DPP-IV was measured at 100 µM, and the compounds that showed favorable activity were selected for further in vitro and in silico testing. A total of four compounds were selected (**4a**, **4e**, **4f**, and **4g**), and their IC_50_ were measured (Supplementary Materials), except for **4e** because it possesses inhibitory activity lower than 50% (Table 1). The most active compound is **4g** with IC_50_ = 0.35 µM.

### 2.2. Geometry Optimization

For all the theoretical computations the Gaussian 16 software package was used [29]. The geometry of the compounds was optimized at the B3LYP/6-31 + G(d,p) level of theory. Harmonic vibrational frequencies were calculated to verify that the optimized structures are true minima on the potential energy surfaces. The two diastereomers with *cis*- and *trans*-relative orientations of the substituents were studied systematically. The effect of the medium was considered, and all computations were performed using IEFPCM formalism [30]. Xylene was used as a solvent to mimic the reaction conditions in the synthetic methodology. The energy difference between *cis*- and *trans*-diastereomers in xylene is presented in Table 2. The *trans*-diastereomer was found to be more stable for all of the studied compounds **4a**–**g**.

**Figure 3:**
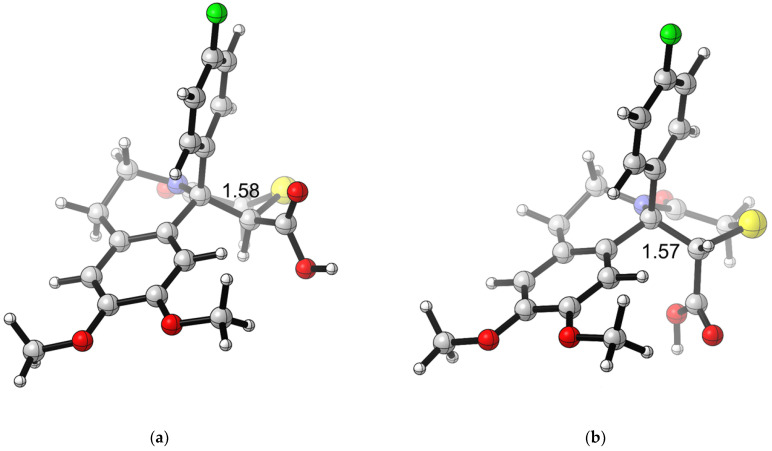
B3LYP/6-31+G(d,p)-optimized geometry for compound **4g**: (**a**) *cis*-**4g** in xylene; (**b**) *trans*-**4g** in xylene. Color scheme: C—gray, O—red, N—blue, H—light gray, S—yellow, F—green. CYLview, 1.0b; Legault, C. Y., Université de Sherbrooke, 2009 (http://www.cylview.org, accessed on 25 October 2024).

The geometry of **4g** optimized at B3LYP/6-31+G(d,p) theory levels in xylene is represented in Figure 3. The geometry of the other compounds from the series shows similar characteristics (Supplementary Materials).

### 2.3. Docking Analysis

**Figure 4:**
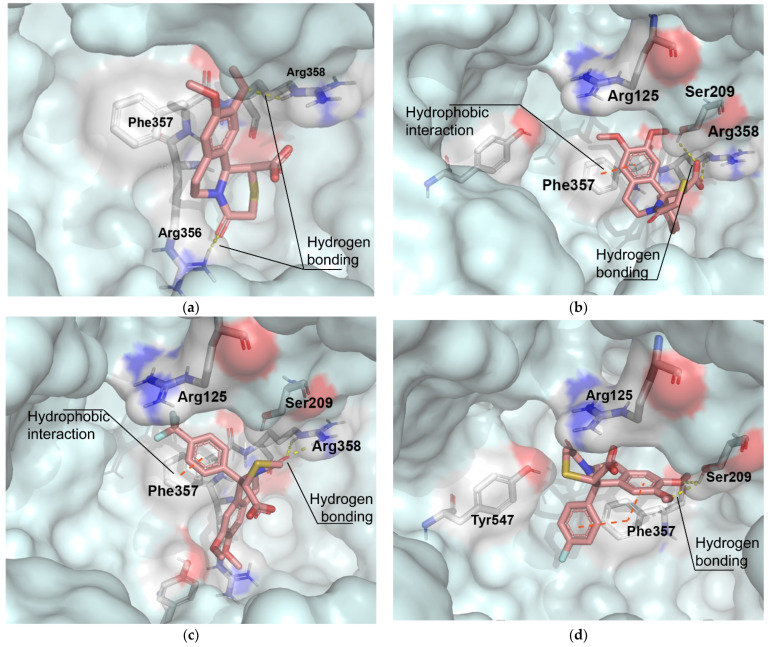
Interaction modes of the energy minimized poses of the most active compounds to the crystal structure of DPP-IV: (**a**) compound **4a**; (**b**) compound **4e**; (**c**) compound **4f**; (**d**) compound **4g**. Color scheme: C—deep salmon, O—red, N—blue, S—yellow, F—light blue.

In order to validate the docking protocol, we did the redocking of the ligand into its cocrystalized structure, and the binding mode of the energy minimized pose was reproduced fairly well compared to the X-ray complex crystal structure reported by Mattei et al. (Supplementary Materials) [25]. The optimized geometry of the ionized form of the *trans*-diastereomers (given by the DFT analysis) was used, since the in vitro DPP-IV screening was performed under physiological conditions. Among the first nine conformers with the most favorable binding affinities that were generated by the docking software (presented in the Supplementary Materials), we selected the lowest energy conformation for our analysis. The binding energies and the type of interaction in our series compounds are listed in Table 3. The interaction modes of the most active compounds **4a**, **4e**, **4f**, and **4g**, optimized in xylene, are shown in Figure 4. The core benzo[a]quinolizidine subunit **4a** (IC_50_ = 41.13 µM) is closer to the S2-extended pocket and forms H-bonds with Arg356 and Arg358. The docking modes of the other compounds show that they are closer to the N-terminal recognition region and the S2-pocket and possesed higher inhibitory activity compared to **4a**.

### 2.4. Acute Toxicity Assay

**Figure 5:**
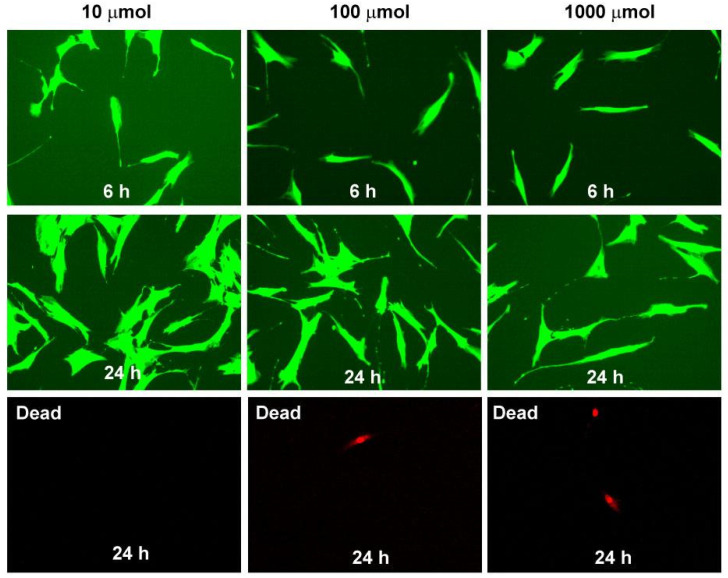
Cytotoxicity evaluation of compound **4g**. Mesenchymal stem cells after 6 and 24 h at different concentrations of **4g** colored in green; dead cells after 24 h colored in red.

In order to evaluate the acute toxicity, we treated cultures of adipose-derived stem cells (ADSCs) to different concentrations of the obtained compounds [31,32]. More than 95% viability ADSCs were reported with all three substances at 6, 24, and 48 h. The obtained results are comparable to the controls. All tested compounds showed no toxicity on ADSCs at doses of 10, 100, and 1000 µmolar at 6, 24, and 48 h (Figure 5).

## 3. Discussion

In an attempt to develop new DPP-IV inhibitors based on previously unexplored heterocyclic ring systems, we developed a synthetic approach for the preparation of aryl-substituted [1,4]thiazino[3,4-a]isoquinolines. The methodology relies on a one-step reaction between substituted cyclic imines and monocyclic enolizable anhydrides [27,28]. At the time our previous report was published, there was no alternative procedure in the literature for the preparation of the compounds **4d**,**e** that feature an angular aryl substituent. Moreover, the reaction proceeds through a stereoselective manner, yielding the *trans*-diastereomer [28].

A preliminary step in the in silico studies is molecular geometry optimization. There are two stereocenters at the heterocyclic core structure (C-1 and C-11b), and the orientation of the substituents there could be *cis*- and *trans*-relative to the heterocyclic ring. The results indicate that the type of the substituent has a limited effect on the energy difference between the two diastereomers (Table 2). The *trans*-diastereomer was found to be more stable in all cases. For compounds **4e**–**4g**, the synthetic pathway that was developed in our lab allowed only one of the products to be isolated after the crystallization procedure, and it is in favor of the *trans*-diastereomer. These results correspond well with the theoretical data (ΔG in the range 3–5 kcal/mol). The only case in which both *cis*- and *trans*-diastereomers were obtained and isolated in equimolar ratio was for compound **4a** [27]. This could be rationalized in terms of the relatively low energy difference between the *cis*- and *trans*-diastereomers for **4a** (0.2 kcal/mol).

Molecular docking experiments were performed for the analysis of the interaction between the examined compounds and the DPP IV enzyme. The most important selectivity cliffs for DPP-IV inhibition by gathering years of published structure–activity relationship (SAR) data from studies of different inhibitor series shows that the Glu205, Glu206, Tyr662, Arg125, and Phe357 residues in the N-terminal recognition region, together with the residues from the S1 and S2 pockets, comprise the most important anchor points for inhibitor recognition in the DPP-IV binding site [33]. An electrostatic interaction with Arg358 may also increase both DPP-IV activity and improve selectivity against DPP8 and DPP9. In our study, the docking analysis revealed H-bonding with Ser 209 and Arg 358. The formation of hydrogen bonds with Arg358, leading to an increase in inhibitory potential has been previously observed [34]. As we can see from Table 3, with the addition of cyclic aromatic moieties on position 11b, the inhibitory activity of **4f** and **4g** increases. This could be due to the presence of π-π interactions with Phe357, which is a crucial amino acid for inhibitory recognition, also studied by other authors [35,36]. The presence of a fluorine atom in the benzene ring of **4g** also makes a significant difference in terms of inhibitory activity. The fluorine atom could participate in additional hydrogen bonds through water molecules with amino acid residues in the active site [37]. Furthermore, the two benzene rings in **4g** adopt a T-shape geometry for Phe357, which could contribute to stronger and tighter hydrophobic interactions. An increase in the inhibitory activity was observed also with all other compounds (**4e**, **4f**) that possess an electronegative substituent in position 4 in the 11b-phenyl ring.

Among the synthesized compounds **4a**–**l**, three of them (**4a**, **4f**, and **4g**) demonstrated IC_50_ in the mid-micromolar range, with **4g** (IC_50_ = 0.35 µM) being the lead compound in the series. These results are comparable to some natural product inhibitors of DPP-IV published recently, which also exhibited IC_50_ values in the micromolar range [38,39]. The heterocyclic fragment in compounds **4a**–**l** is rarely found in the recent literature, which makes them interesting from a synthetic and medicinal chemical point of view. Our results can also be compared to other DPP-IV inhibitors from the recent literature in terms of inhibitory potential [40,41,42].

Furthermore, these compounds did not exhibit any cytotoxicity towards mesenchymal stem cells even at a 1000 µM concentration, making them suitable candidates for further in vitro and in vivo studies.

## 4. Materials and Methods

### 4.1. Synthesis of Target Compounds ***4a**–**g***

The compounds **4a**–**g** were synthesized using a reaction between 3,4-dihydroisoquinoline derivatives **5** and thiodiacetic anhydride **6** [27,28]. The necessary starting compounds **5** were prepared using methods described in the literature [43,44,45,46]. The anhydride **6** was obtained by the TFAA-mediated cyclization reaction of the corresponding 2,2′-thiodiactic acid in anhydrous dichloromethane [47].

### 4.2. DPP-IV Inhibitory Assay

The effect of the compounds on the activity of human DPP-IV was determined by the cleavage rate of 7-Amino-4-methylcoumarin (AMC). The assay was performed according to the manufacturer’s protocol (SensoLyte AMC DPP-IV Assay Kit, AnaSpec, Fremont, CA, USA, Cat. No AS-72197). The assay was performed in a black, flat bottom, 96-well microplate with non-binding surface. Preincubation of 20 µL DPP-IV (0.25 µg/mL) or an assay buffer with different concentrations of tested substances (0.01 µM, 0.05 µM, 0.1 µM, 0.5 µM, 1 µM, 2 µM, 12.5 µM, 25 µM, 50 µM, 75 µM, 100 µM, 150 µM, 200 µM, 250 µM, and 300 µM) was performed for ten minutes at 25 °C. Sitagliptin was used as a positive control inhibitor. After mixing the reagents, as described in the manufacturer’s protocol, the fluorescence (excitation at 380 nm and emission at 460 nm) of cleaved AMC was measured in 5 min intervals for 30 min using a multimode plate reader Mithras LB 943 (Berthold Technologies GmbH & Co. KG, Bad Wildbad, Germany). The DPP-IV activity was obtained as relative fluorescent units (RFU). A plot of the fluorescence versus the time was obtained for each of the tested concentrations, and then the relative inhibition (expressed in percentage) was calculated. Initially, a screening of all compounds at 100 µM was performed to eliminate the compounds with low inhibitory activity. After that, the remaining compounds were tested at the full concentration range [48]. The sigmoidal dose–response fitting tool in GraphPad Prism (version 10.3.0) was used to determine IC_50_ values from the obtained experimental data.

### 4.3. Molecular Docking

For the purpose of our investigation, we used AutoDock Vina 1.1.2 [49]. The preparation of the *pdbqt* files of the ligands and the receptor as well as the determination of the grid box size were carried out using Autodock Tools (ADT) version 1.5.6. The grid box was built around the substrate binding site residues with the space kept between the grid points 1.000 Å, with an exhaustiveness value of 8 [50,51,52]. In the configuration files, the grid was set to be 20 Å × 20 Å × 20 Å, and the coordinates along x, y, z were centered at 68.086 Å (x), 72.879 Å (y), and 71.424 Å (z), respectively. The docking experiment was carried out using the default parameters of the automatic settings to set the genetic algorithm parameters. The docked conformation with the most favorable docking score was selected to analyze the binding mode. When the dockings were completed, the docking results were visualized in 3D using the PyMOL molecular graphic system (version 3.0). The molecular docking studies were performed by keeping the receptor molecules rigid and the molecules of the synthesized compounds flexible. Through this method, we obtained the binding modes of the most energetically favorable ligand–receptor complexes. The crystallographic structure of human DPP IV was downloaded from Protein Data Bank (PDB) with the following code: 3KWF. To prepare the enzyme for docking, all water molecules and unnecessary heteroatoms were removed, and the polar hydrogens were added.

### 4.4. Cytotoxicity Analysis

The acute toxicity of compounds **4a**–**g** was tested by measuring their effects on adipose tissue-derived stem cells viability. ADSCs (2 × 10^4^) cultured in 24-well plates were treated with each of the analyzed compounds for 6, 24, and 48 h before being subjected to assays. The compounds were tested at concentrations of 10, 100, and 1000 µmol. The Cellstain double kit, containing Calcein-AM and Propidium Iodide (PI) solutions, staining viable, and dead cells, was used. The calcein generated from Calcein-AM in a viable cell emits green fluorescence (excitation: 490 nm, emission: 515 nm). PI passes through a disordered cell membrane and intercalates with the DNA double helix to emit red fluorescence (excitation: 535 nm, emission: 617 nm). A fluorescence microscope (20×) was used for detection and image generation.

## 5. Conclusions

To identify the biological potential of the synthesized compounds, we carried out an in vitro DPP-IV inhibition assay, and the SAR properties of the most potent compounds were further evaluated through geometry optimization and molecular docking. The DFT computations for the relative stability of the *cis*- and *trans*-diastereomers is in agreement with the experimental data. The most active compound is **4g** with IC_50_ = 0.35 µM. All of the molecules are considered as non-cytotoxic because the percentage of the cell viability is higher than 95%. The molecular docking analysis of our leading compounds shows that they are close to the most important selectivity cliffs for DPP IV inhibition. These results provide a necessary basis for further structural–activity optimization and drug research.

## Figures and Tables

**Table 1:** Inhibitory activity (%) and IC_50_ (in µM**)** values of compounds **4a**–**4l**.

Entry	R	DPP-IV Inhibitory Activity, % *	IC_50_, µM	Entry	R	DPP-IV Inhibitory Activity, % *	IC_50_, µM
**4a**	H	70	41.13	**4g**	*p*-FC_6_H*4*	96	0.35
**4b**	CH_3_	n/a	n/a	**4h**	*m*-Cl	n/a	n/a
**4c**	CH_2_CH_3_	n/a	n/a	**4i**	*m*-Br	n/a	n/a
**4d**	C_6_H_5_	n/a	n/a	**4j**	*p*-OCH_3_	n/a	n/a
**4e**	*p*-BrC_6_H_4_	9	n/a	**4k**	*p*-NO_2_	n/a	n/a
**4f**	*p*-F_3_CC_6_H_4_	88	15.91	**4l**	*p*-CN	n/a	n/a

* Measured at 100 µM for each compound. n/a—no inhibitory activity.

**Table 2:** Relative energy differences between diastereomers optimized at the B3LYP/6-31+G(d,p) theory level (in kcal/mol).

General Formula	Entry	R	∆G_(trans-cis)_ ^1^, kcal/mol
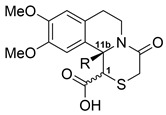	**4a**	H	0.2
	**4e**	*p*-BrC_6_H_4_	4.0
	**4f**	*p*-F_3_CC_6_H_4_	3.7
	**4g**	*p*-FC_6_H*4*	4.3

^1^ in xylene, protonated.

**Table 3:** Structure–activity relationship of compounds.

Entry	Relative Stereochemistry	Binding Energy, kcal/mol	IC_50_, µM	Residues Involved in H-Bonds	Hydrophobic Interactions/π-π Interactions
**4a**	*rel*-(1*R*,11b*S*)	−7.0	43.13	Arg356, Arg358	—
**4e**	*rel*-(1*S*,11b*S*)	−7.0	>100	Ser209, Arg358	Phe357
**4f**	*rel*-(1*S*,11b*S*)	−7.2	15.91	Arg358	Phe357
**4g**	*rel*-(1*S*,11b*S*)	−7.8	0.35	Ser209	Phe357

## Data Availability

The original contributions presented in this study are included in the article/Supplementary Materials. Further inquiries can be directed to the corresponding author/s.

## Supplementary Materials

The following supporting information can be downloaded at: https://www.mdpi.com/article/10.3390/ijms252111753/s1.
