# Supplementary material for: Angular-Substituted [1,4]Thiazino[3,4-a]Isoquinolines: Biological Evaluation and In Silico Studies on DPP-IV Inhibition
Source: Int J Mol Sci. 2024 Nov 1;25(21):11753. doi: 10.3390/ijms252111753 (PMC11545999; doi:10.3390/ijms252111753)
Supplement: Supplementary file 1 [file ijms-25-11753-s001.zip › ijms-3281050-supplementary.pdf]

Supplementary Materials

for

Angular-substituted [1,4]thiazino[3,4-a]isoquinolines: biological evaluation and *in silico* studies toward DPP-IV inhibition

Aleksandar Pashev [1*], Valentin Petrov [1], Aleksandrina Pesheva [1], Lidiya Petrova [2], Kalina Ilieva [2], Galya Stavreva [1], Milena Atanasova [2], Diana Cheshmedzhieva [3], George Altankov [4] and Teodora Aleksandrova [1]

[1] Faculty of Pharmacy, Medical University Pleven, 1. St. Kliment Ohridski str., 5800 Pleven, Bulgaria;

[2] Faculty of Medicine, Medical University Pleven, 1. St. Kliment Ohridski str., 5800 Pleven, Bulgaria;

[3] Faculty of Chemistry and Pharmacy, Sofia University "St. Kliment Ohridski", 1. James Bourchier blvd., 1164 Sofia, Bulgaria;

[4] Research Institute, Medical University Pleven, Pleven, 5800, Bulgaria;

* Correspondence: aleksandar.pashev@mu-pleven.bg;

1. Experimental determination of IC_{50} values

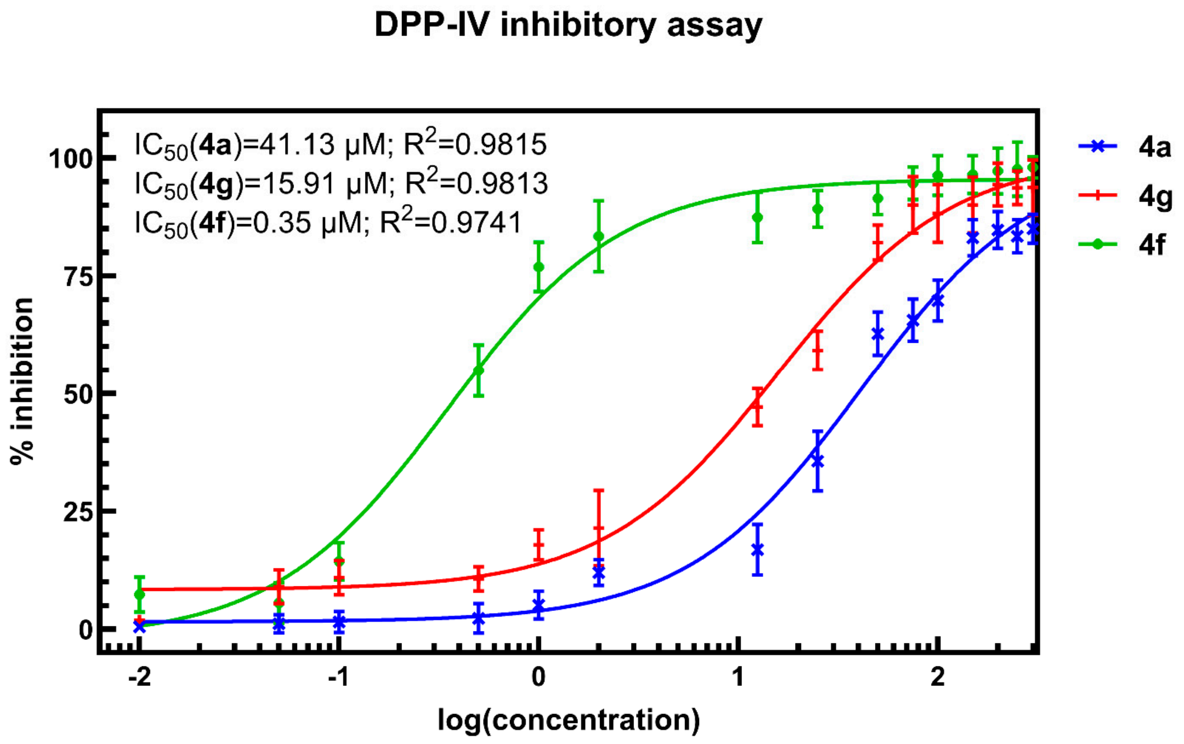

Figure S1. Inhibition of DPP-IV vs. log(concentration) for compounds **4a**, **4g**, and **4f**

2. Geometry optimization data

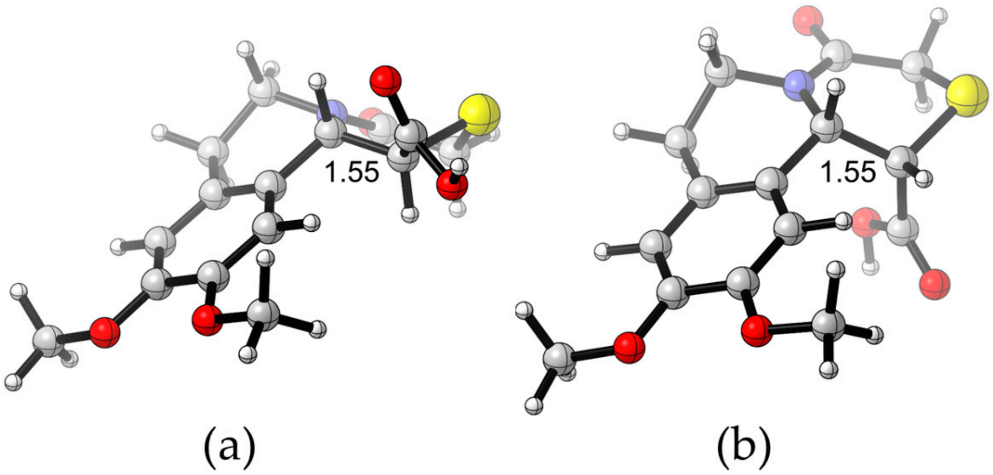

Figure S2. Optimized geometry for compound **4a**: (a) *cis*-**4a** in xylene; (b) *trans*-**4a** in xylene. C – grey, O – red, N — blue, H – light grey, S – yellow. CYLview, 1.0b; Legault, C. Y., Université de Sherbrooke, 2009 (http://www.cylview.org)

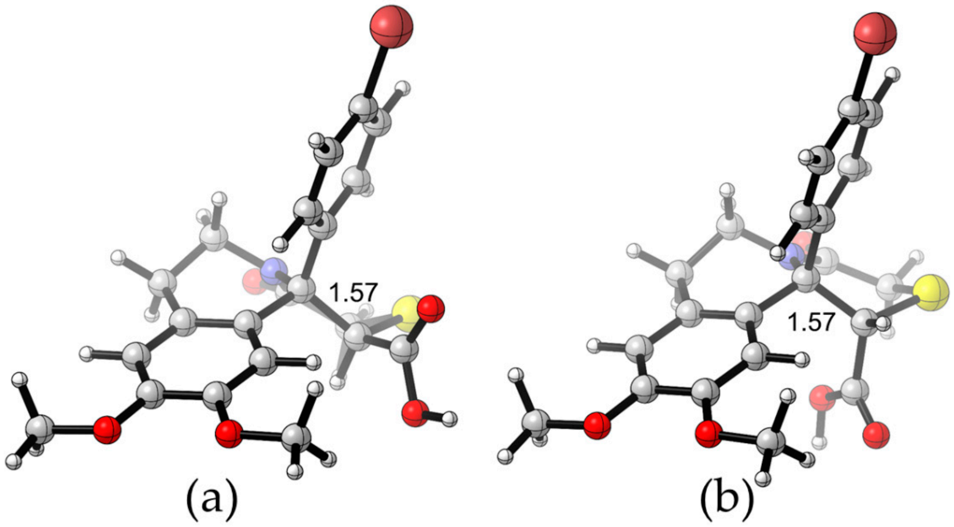

Figure S3. Optimized geometry for compound **4e**: (a) *cis*-**4e** in xylene; (b) *trans*-**4e** in xylene. Color scheme: C – grey, O – red, N — blue, H – light grey, S – yellow, Br – maroon.

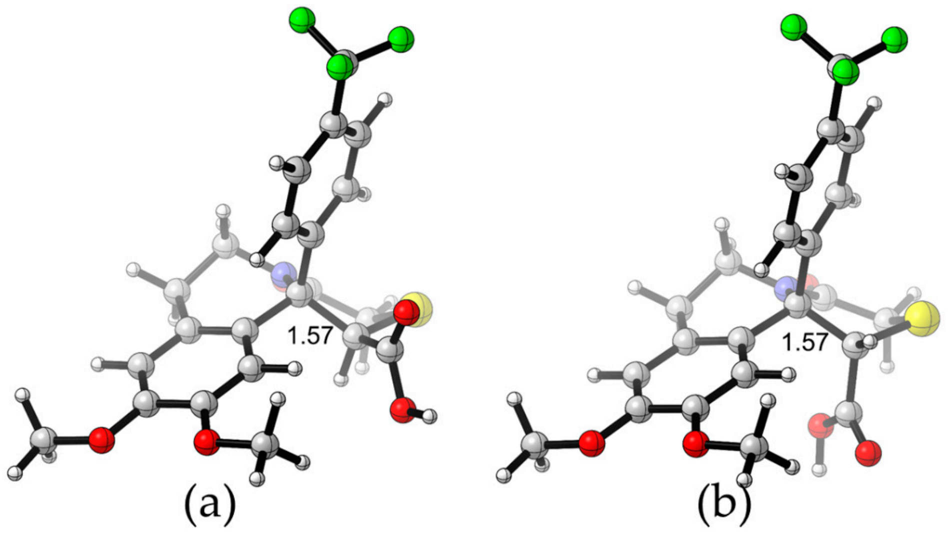

Figure S4. Optimized geometry for compound **4f**: (a) *cis*-**4f** in xylene; (b) *trans*-**4f** in xylene. Color scheme: C – grey, O – red, N — blue, H – light grey, S – yellow, F – green.

Optimized Cartesian coordinates, Electronic energies, and the imaginary frequencies (all in atomic units). The computations are performed at B3LYP/6-31+G(d,p) level of theory.

(a) *cis*-**4a**

0 1
C,0,3.7863188022,-1.6143267757,-0.6653846911
C,0,3.1985032538,-1.4977661083,0.6189549373
C,0,2.1268327478,-0.6290141923,0.7999403225
C,0,1.6056789099,0.1459931501,-0.2581214053
C,0,2.179835662,0.017538346,-1.5288094534
C,0,3.2665844026,-0.8586149739,-1.7100936004
N,0,0.3201367988,2.0610698513,-1.1257512783
C,0,0.3030396003,1.4130440243,-2.4411478946
C,0,1.6731874673,0.802522674,-2.7232198769
C,0,0.3672282195,1.6757531744,1.3930802762
S,0,-0.6085844098,3.2412731541,1.490289805
C,0,0.4489798404,4.0987418675,0.2812501473
C,0,0.4768777121,3.4143768824,-1.0749422806
O,0,4.8320478095,-2.4807384298,-0.7684465738
O,0,3.7506720077,-2.2697490648,1.5986845901
C,0,5.4562426543,-2.6372724218,-2.040385498
C,0,3.1899451216,-2.2105596075,2.9067138976
O,0,0.6090793831,4.1058114811,-2.0908549817
C,0,-0.2834849779,0.7260888963,2.3914796559
O,0,-1.1924314166,-0.0383914344,2.1457911338
O,0,0.2561079413,0.8593394099,3.6217938956
H,0,1.6992679782,-0.5591374395,1.7902242526
H,0,3.6991937241,-0.9398441933,-2.7011246456

(b) *trans*-**4a**

0 1
C,0,3.4637923458,-2.0534764784,-0.5879086892
C,0,2.7343002271,-2.0048786661,0.628981263
C,0,1.7489573294,-1.0375888695,0.7908487443
C,0,1.4619309,-0.0959759743,-0.2210017614
C,0,2.1571817826,-0.1665246602,-1.4287127157
C,0,3.15543238,-1.1451185339,-1.5946315932
N,0,0.18228072,1.8569589517,-1.1042511532
C,0,0.3468643163,1.2119418277,-2.4152903464
C,0,1.7909988587,0.7493070103,-2.5732006435
C,0,0.6282035922,1.6697123049,1.3917562284
S,0,-0.6624500673,2.9262246386,1.7345488666
C,0,-0.4397596603,3.9110076629,0.2154346943
C,0,-0.2320826397,3.158409564,-1.0907672342
O,0,4.4161284247,-3.0227062947,-0.6729707803
O,0,3.0701370277,-2.9410674046,1.560695559
C,0,5.178747279,-3.1165641549,-1.8736026595
C,0,2.3922078679,-2.923819157,2.8143319221
O,0,-0.4132130288,3.802234669,-2.1310850016
C,0,2.0242864344,2.2341463074,1.6184383684
O,0,2.6634272021,2.0557628167,2.6354666067
O,0,2.4762908084,2.9850524564,0.591759279
H,0,1.1909677581,-1.0153724683,1.7188008294
H,0,3.681196466,-1.1884882736,-2.5418814485

H,0,0.031926671,2.1572856571,-3.1882788769
H,0,-0.469604418,0.6351623805,-2.4170081174
H,0,1.6151124307,0.1522704966,-3.6043338249
H,0,2.3788552883,1.6065956328,-2.9705041807
H,0,1.3758329808,1.9126964477,1.7415619167
H,0,0.0421550866,5.0996246149,0.1302984291
H,0,1.4659925795,4.2012490674,0.6769598721
H,0,6.2535825136,-3.3653451329,-1.8894760516
H,0,4.7489378117,-3.0194347534,-2.7862395601
H,0,5.8864381092,-1.6917567903,-2.3918993269
H,0,3.7749119661,-2.9054388517,3.5098186361
H,0,3.2715114069,-1.2024211442,3.3312050663
H,0,2.1391393202,-2.5256987285,2.9041491215
H,0,-0.2403888679,0.2849071621,4.2312535576
C,0,0.3915575245,1.0714916077,-0.0296270405
H,0,-0.5245716751,0.4716772442,-0.1144880047

E=-1410.958959
Nimag=0

H,0,0.0638783228,1.9357724151,-3.1761081009
H,0,-0.3356668174,0.3533805327,-2.4740619383
H,0,1.9074394477,0.2230030138,-3.5276499268
H,0,2.4489863299,1.6275342153,-2.6079369426
H,0,0.4884474876,0.9402748621,2.1892223199
H,0,-1.3445782818,4.5091381059,0.0940620149
H,0,0.4018687926,4.5995526805,0.3301357235
H,0,5.876207504,-3.9395116994,-1.7159838407
H,0,4.5385685153,-3.3404341754,-2.7354683897
H,0,5.7390368894,-2.1928837966,-2.0618776849
H,0,2.8290676583,-3.7357534171,3.3961862654
H,0,2.5489783047,-1.9733058387,3.3380346735
H,0,1.3173359409,-3.104131688,2.6890735195
H,0,3.3602865431,3.3144706997,0.8326511539
C,0,0.3681082178,0.9502609506,0.0488114075
H,0,-0.5819609793,0.4141635955,0.1990279609

E=-1410.962361
Nimag=0

(a) *cis*-**4e**

0 1
C,0,-7.1974791909,1.9128643746,-0.5643753348
C,0,-7.2594356594,0.5056664114,-0.40374401
C,0,-6.0775639714,-0.2157314449,-0.2814940211
C,0,-4.8065928222,0.4020421901,-0.3159827221
C,0,-4.7537000167,1.7949636291,-0.4503016054
C,0,-5.9511689429,2.5261186556,-0.5765274262
C,0,-3.5172744733,-0.4651054119,-0.2024954389
N,0,-2.3654676797,0.3879853812,-0.64358046
C,0,-2.3189551204,1.6778481839,0.0654596593
C,0,-3.4463393228,2.5587109044,-0.4520812384
C,0,-3.667884833,-1.6829354232,-1.1895728874
S,0,-2.0658066204,-2.4820343291,-1.6333530881
C,0,-1.4928883205,-1.0158416069,-2.5390805922
C,0,-1.5399863354,0.2699723457,-1.7312029195
O,0,-8.3905512212,2.5550563903,-0.6845595363
O,0,-8.5101572934,-0.035842064,-0.3760977243
C,0,-8.3819383339,3.9717097688,-0.8497628055
C,0,-8.6380782259,-1.4464530231,-0.2181399404
O,0,-0.7912295688,1.1889556807,-2.0810607452
C,0,-4.5488933726,-2.8469333068,-0.7538192733
C,0,-3.2272613244,-0.9074750068,1.2570073499
O,0,-4.5094032126,-3.4265943192,0.3093567536
O,0,-5.3652028301,-3.2268717511,-1.7654713013
C,0,-1.9499121738,-1.3745295659,1.6030620993
C,0,-1.6548182207,-1.7897199276,2.9011468004
C,0,-2.6480644886,-1.7271303829,3.876064919
C,0,-3.918415478,-1.2488213397,3.5713054761

(b) *trans*-**4e**

0 1
C,0,-7.2063785277,1.729891502,-0.3673409011
C,0,-7.2130304204,0.3158594272,-0.2640010733
C,0,-6.0028692122,-0.3628922259,-0.1735352702
C,0,-4.7592109165,0.3099857231,-0.1905254519
C,0,-4.7593600279,1.703504323,-0.3004202481
C,0,-5.9842961389,2.3919557854,-0.3831595484
C,0,-3.4440293414,-0.5142150051,-0.1074027776
N,0,-2.3108047736,0.3536986106,-0.5207120208
C,0,-2.3098093478,1.66188438,0.15589322
C,0,-3.4737097296,2.4970055874,-0.3544341544
C,0,-3.6038051887,-1.7767334484,-1.0273306045
S,0,-2.0272695801,-2.6480895164,-1.3604994392
C,0,-1.1486934432,-1.2337762547,-2.102945967
C,0,-1.3048349359,0.114016449,-1.4158737342
O,0,-8.4248238095,2.3301143811,-0.4446785861
O,0,-8.439873683,-0.276780575,-0.2616707756
C,0,-8.4725883704,3.7512320934,-0.5536733526
C,0,-8.5078083234,-1.7012331182,-0.2154978978
O,0,-0.4731381006,0.9812761759,-1.7067178496
C,0,-4.3412129327,-1.5890987262,-2.350246511
C,0,-3.1609289001,-1.0113072002,1.3457250085
O,0,-5.2067383525,-2.3446675931,-2.7451227367
O,0,-3.9030948999,-0.536383252,-3.0710739139
C,0,-1.8460137834,-1.2984382331,1.7444548482
C,0,-1.5573406146,-1.7836435568,3.0202782419
C,0,-2.5999070397,-1.9852515396,3.9214361052
C,0,-3.9141662823,-1.6976803925,3.5654540873

C,0,-4.1958322643,-0.8387768599,2.2655728031
Br,0,-2.2551836033,-2.2827923162,5.6603319348
H,0,-6.1582597951,-1.2818343609,-0.1317202213
H,0,-5.8816142448,3.6030960473,-0.6819494523
H,0,-1.342140059,2.1265239251,-0.1032708893
H,0,-2.4392437995,1.4802621952,1.1336888878
H,0,-3.5365725682,3.4569965404,0.170155214
H,0,-3.2043455396,2.8987628829,-1.4674858276
H,0,-4.0816155107,-1.2990280875,-2.1247752684
H,0,-0.4511780943,-1.1751734015,-2.8224579396
H,0,-2.0738709429,-0.8858567206,-3.4592169391
H,0,-9.4288400882,4.2647013254,-0.9305616196
H,0,-7.9286150043,4.4697139064,0.015559701
H,0,-7.8488426011,4.2617434099,-1.762983588
H,0,-9.7093871102,-1.6480548153,-0.23238547
H,0,-8.1540757937,-1.9837473626,-1.0424883079
H,0,-8.2181839499,-1.7810968061,0.7383831233
H,0,-5.8284309421,-4.0354976517,-1.4839522516
H,0,-1.1671063595,-1.4136959949,0.8564726758
H,0,-0.6621292426,-2.1503884692,3.1464360002
H,0,-4.6841677126,-1.1869878016,4.3363659774
H,0,-5.1864427214,-0.4587415975,2.0495804697

E=-4213.135314
Nimag=0

C,0,-4.1833242609,-1.2102162238,2.2840365291
Br,0,-2.2211300237,-2.6544633098,5.6688846375
H,0,-6.0334817584,-1.4412201635,-0.099412217
H,0,-5.9579185883,3.4728944349,-0.4646585624
H,0,-1.3519406647,2.1395544247,-0.0374257396
H,0,-2.4080562652,1.4874622902,1.2326772064
H,0,-3.5658867527,3.409618231,0.2462014427
H,0,-3.2700735653,2.8137579416,-1.3860065035
H,0,-4.191081392,-2.511039942,-0.4764951206
H,0,-0.0839456106,-1.4740266516,-2.0764370438
H,0,-1.4358204685,-1.1091264383,-3.1498108944
H,0,-9.5311504712,4.0071259438,-0.602184888
H,0,-8.0196405335,4.232131701,0.3214701883
H,0,-7.9704396678,4.0972117208,-1.4650125947
H,0,-9.5694721827,-1.9453931513,-0.2568882161
H,0,-7.9935089139,-2.1523357576,-1.0716824998
H,0,-8.0831784876,-2.090035981,0.718260324
H,0,-4.4137339287,-0.5109189204,-3.8995790392
H,0,-1.0220529248,-1.1339131921,1.0619628003
H,0,-0.5327220731,-1.9970471083,3.3033074796
H,0,-4.7225317887,-1.8395407184,4.2739872273
H,0,-5.2099300015,-0.9707169312,2.0384267864

E=-4213.142769
Nimag=0

(a) *cis*-**4f**

0 1
C,0,-7.2146714446,2.0256761229,-0.8944435522
C,0,-7.270010561,0.6180077612,-0.733817073
C,0,-6.0846992938,-0.098010988,-0.6120097379
C,0,-4.8165460807,0.5264104145,-0.6431315942
C,0,-4.770582334,1.9197885331,-0.7760605565
C,0,-5.9713737057,2.6449086875,-0.9051794921
C,0,-3.5220014951,-0.333771735,-0.5300338304
N,0,-2.3742470144,0.5257134434,-0.9660983518
C,0,-2.3363609335,1.8155948898,-0.2563308894
C,0,-3.4676577877,2.6908351443,-0.7741969267
C,0,-3.6637351584,-1.5502295697,-1.5198294025
S,0,-2.0566227328,-2.3417192155,-1.9593070526
C,0,-1.4853300828,-0.8717826327,-2.8600830959
C,0,-1.5446754003,0.4134357268,-2.052232575
O,0,-8.4105014388,2.6617222561,-1.0153620683
O,0,-8.5182189222,0.0716078311,-0.7058014194
C,0,-8.4096645016,4.0790676064,-1.1764440145
C,0,-8.6419694932,-1.3391017271,-0.5454639976
O,0,-0.8021951457,1.3379704674,-2.3992293075
C,0,-4.5417653271,-2.7181579715,-1.0897791979
C,0,-3.2293850424,-0.7788106841,0.9307245936

(a) *trans*-**4f**

0 1
C,0,-7.2191132331,1.8569039791,-0.7125411481
C,0,-7.2193309435,0.4432627919,-0.6015045976
C,0,-6.0061762166,-0.2292642325,-0.5044900128
C,0,-4.7657077083,0.4496788195,-0.523212587
C,0,-4.7720079279,1.8427549719,-0.6408949774
C,0,-6.000233037,2.5249372059,-0.7301526102
C,0,-3.4461527596,-0.3674238025,-0.4322645434
N,0,-2.3163157877,0.5022116201,-0.8491775059
C,0,-2.3222472694,1.8159056242,-0.1829654715
C,0,-3.4900374307,2.6417051852,-0.6992443846
C,0,-3.5977269881,-1.6390094183,-1.3411967508
S,0,-2.0154797733,-2.502591295,-1.6677074432
C,0,-1.1471131425,-1.0887794036,-2.4233288739
C,0,-1.3119756843,0.2635653458,-1.7475840221
O,0,-8.4401294876,2.4501466902,-0.7961326448
O,0,-8.4431350822,-0.1548625152,-0.5991477483
C,0,-8.4956947023,3.8701822182,-0.9172560332
C,0,-8.5053409715,-1.5793276866,-0.5429244134
O,0,-0.488968347,1.1352945748,-2.0477377969
C,0,-4.336446515,-1.4647297385,-2.6656635085
C,0,-3.1626444016,-0.8533241772,1.0263447776

O,0,-4.5166371068,-3.2855267234,-0.0193170289
O,0,-5.3348986498,-3.1156226559,-2.1119374616
C,0,-1.9448956775,-1.2281938221,1.2770793225
C,0,-1.6493831487,-1.6378983466,2.5750105405
C,0,-2.6387620467,-1.5959681527,3.5623680225
C,0,-3.9147719311,-1.1300500745,3.241580603
C,0,-4.2011665395,-0.7243513695,1.9375553328
C,0,-2.3051060212,-1.9872908551,4.9743603035
F,0,-1.4017138337,-2.9994043299,5.0323643205
F,0,-1.7586799079,-0.9515833465,5.6775362536
F,0,-3.394793556,-2.3884818652,5.6764291776
H,0,-6.1608437287,-1.1646827146,-0.4630689353
H,0,-5.9067623064,3.7221849492,-1.0104818415
H,0,-1.3615891148,2.2694333336,-0.4227321253
H,0,-2.4569493593,1.6173579035,0.811862092
H,0,-3.5638509375,3.5873064717,-0.1503233271
H,0,-3.2260251975,3.0343083248,-1.7884900152
H,0,-4.0758533896,-1.1646461234,-2.4550941835
H,0,-0.4407750217,-1.0257245594,-3.136004039
H,0,-2.059950606,-0.7442387844,-3.7845321321
H,0,-9.4581276633,4.3662746592,-1.2573321511
H,0,-7.9599509557,4.5768557345,-0.309153504
H,0,-7.8775643847,4.3746434117,-2.0883960378
H,0,-9.7128175621,-1.5432542285,-0.5549853668
H,0,-8.1606098085,-1.876043316,-1.3715116135
H,0,-8.2173452249,-1.6717258265,0.4096606646
H,0,-5.7951939585,-3.9265105581,-1.8320096882
H,0,-1.1631117799,-1.2560992632,0.5295144672
H,0,-0.6510302442,-1.9883956088,2.8145780498
H,0,-4.6871680995,-1.0814431707,4.0013362286
H,0,-5.1966793427,-0.3603794542,1.718242614

E=-1979.066642
Nimag=0

O,0,-5.196326462,-2.2290686389,-3.0553339991
O,0,-3.9046753557,-0.4141252111,-3.3928673071
C,0,-1.8455265578,-1.1259153435,1.4298243757
C,0,-1.5605227482,-1.5938375113,2.7113107263
C,0,-2.5979009691,-1.8021721716,3.6242600149
C,0,-3.9133358698,-1.5250887875,3.2475609852
C,0,-4.1871264181,-1.053043748,1.9628524707
C,0,-2.2906856097,-2.2581376275,5.0236343891
F,0,-1.199106656,-3.0627137763,5.0784627807
F,0,-2.0336112256,-1.2093131591,5.8579530045
F,0,-3.3186337543,-2.9453416422,5.582910364
H,0,-6.0324213172,-1.3073228825,-0.4245645469
H,0,-5.9789820013,3.6054626432,-0.8182701112
H,0,-1.3666389531,2.2965444535,-0.3800644801
H,0,-2.4191747117,1.6499015297,0.8953614501
H,0,-3.5865991748,3.557937839,-0.104962849
H,0,-3.2874583243,2.9522533312,-1.7328772911
H,0,-4.1812188807,-2.3728194358,-0.7855284129
H,0,-0.0807763176,-1.3217774303,-2.3952835613
H,0,-1.4356243028,-0.9754244629,-3.4710426246
H,0,-9.5556060573,4.1195123021,-0.969734345
H,0,-8.0471001499,4.3609868708,-0.045404347
H,0,-7.9939194602,4.2109406936,-1.8307268811
H,0,-9.5658182928,-1.8281011945,-0.5863437677
H,0,-7.9861527834,-2.0344073848,-1.3940305459
H,0,-8.0826307052,-1.9595076858,0.3952791744
H,0,-4.4144545463,-0.3970714743,-4.2221613245
H,0,-1.0233904884,-0.9619493943,0.7456285184
H,0,-0.5330321073,-1.7975689304,2.9929608641
H,0,-4.7265889633,-1.6693919319,3.9505477567
H,0,-5.2144144268,-0.8266785961,1.7094308157

E=-1979.073781
Nimag=0

3. Molecular docking

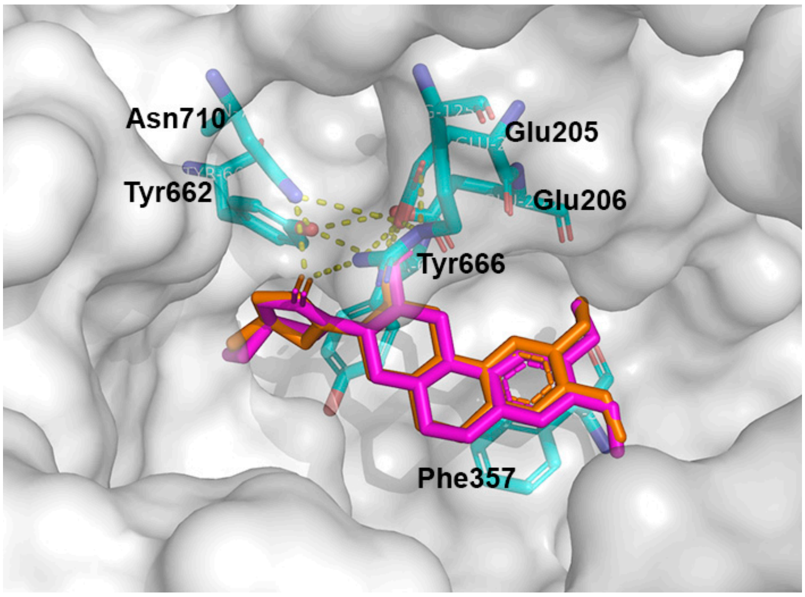

Figure S5. Superimposed structures of the re-docked pose (magenta) and the co-crystallized ligand (orange) in the DPP-IV active site (PDB code: 3KWF).

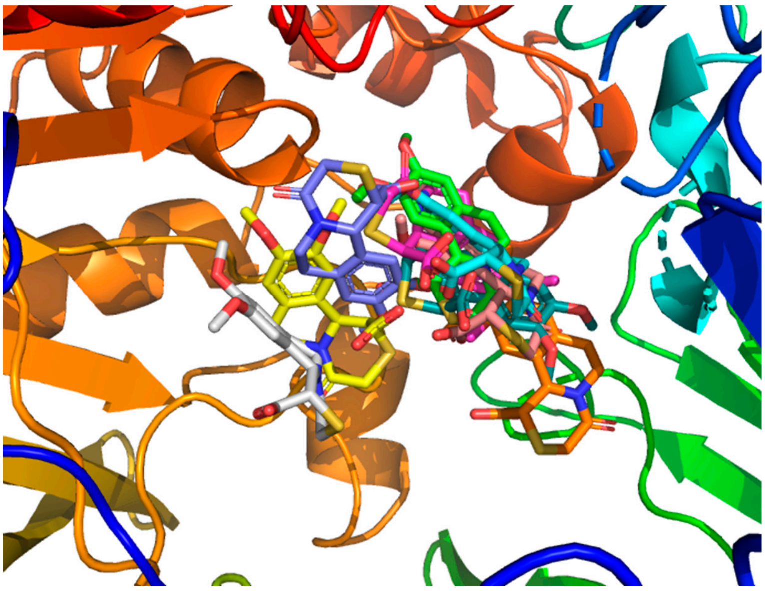

Figure S6. Superimposed structures of the lowest energy poses of **4a** in the active site of DPP-IV (PDB code: 3KWF).

Table S1. The lowest-energy poses of **4a** from the output analysis of the molecular docking.

Binding mode	Binding affinity (kcal/mol)	Residues involved in H-bonds	Hydrophobic interactions/ π-π interactions
1	-7.0	Arg356; Arg358	—
2	-6.5	Tyr752	Trp 629
3	-6.4	Arg61	—
4	-6.1	Ser209	Phe357
5	-6.1	—	—
6	-6.1	Tyr752	His748
7	-6.1	—	Phe357
8	-6.0	—	—
9	-5.8	—	—

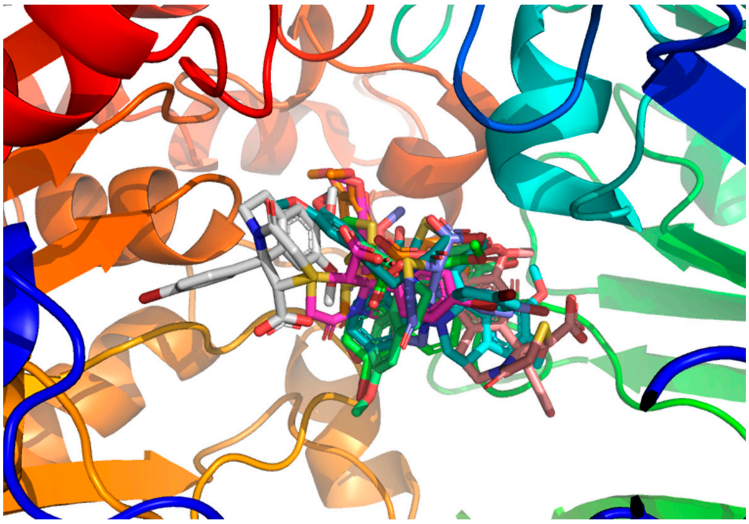

Figure S7. Superimposed structures of the lowest energy poses of **4e** in the active site of DPP-IV (PDB code: 3KWF).

Table S2. The lowest-energy poses of **4e** from the output analysis of the molecular docking.

Binding mode	Binding affinity (kcal/mol)	Residues involved in H-bonds	Hydrophobic interactions/ π-π interactions
1	-7.0	Ser209; Arg358	Phe357
2	-6.3	Tyr547	—
3	-6.2	Tyr547	—
4	-6.0	—	Phe357
5	-6.0	Tyr547	Tyr547
6	-6.0	Ser630	Tyr585
7	-6.0	Ser209	—
8	-5.9	—	—
9	-5.9	Arg125	—

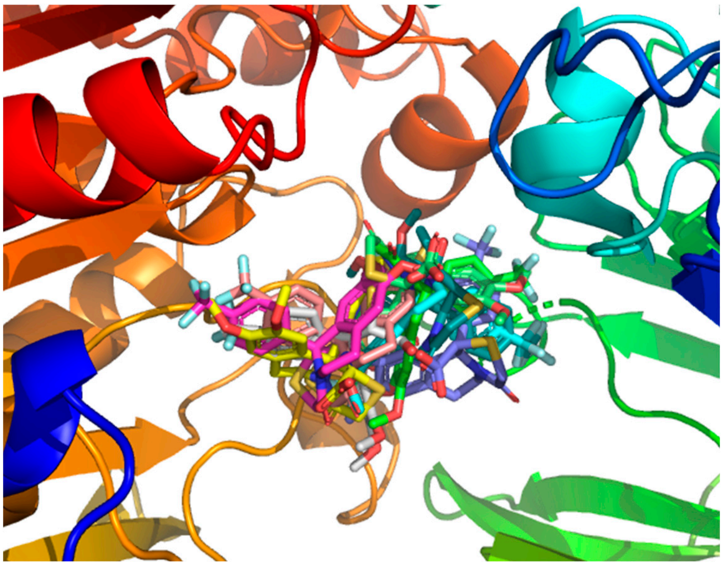

Figure S8. Superimposed structures of the lowest energy poses of **4f** in the active site of DPP-IV (PDB code: 3KWF).

Table S3. The lowest-energy poses of **4f** from the output analysis of the molecular docking.

Binding mode	Binding affinity (kcal/mol)	Residues involved in H-bonds	Hydrophobic interactions/ π-π interactions
1	-7.2	Arg358	Phe357
2	-7.1	Tyr547	Tyr666
3	-7.0	—	Phe357
4	-7.0	—	Phe357
5	-6.9	Tyr547	—
6	-6.8	Ser552	—
7	-6.6	—	Tyr547
8	-6.5	—	Tyr547; Tyr585
9	-5.9	—	Phe357; Tyr585

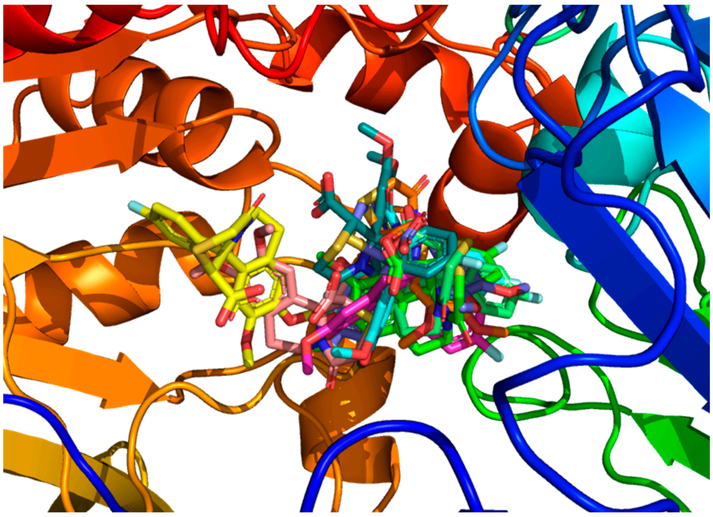

Figure S9. Superimposed structures of the lowest energy poses of **4g** in the active site of DPP-IV (PDB code: 3KWF).

Table S4. The lowest-energy poses of **4g** from the output analysis of the molecular docking.

Binding mode	Binding affinity (kcal/mol)	Residues involved in H-bonds	Hydrophobic interactions/ π-π interactions
1	-7.8	Ser209	Phe357
2	-7.5	—	Phe357
3	-6.9	Tyr547	Phe357
4	-6.6	Tyr662	—
5	-6.5	—	Phe357; Tyr666
6	-6.5	—	Phe357
7	-6.4	—	—
8	-6.4	—	Tyr547
9	-6.4	—	Tyr547
